# Cationic PEGylated polycaprolactone nanoparticles carrying post-operation docetaxel for glioma treatment

**DOI:** 10.3762/bjnano.8.144

**Published:** 2017-07-12

**Authors:** Cem Varan, Erem Bilensoy

**Affiliations:** 1 Department of Nanotechnology and Nanomedicine, Graduate School of Science and Engineering, Hacettepe University, Ankara, 06800, Turkey; 2 Department of Pharmaceutical Technology, Faculty of Pharmacy, Hacettepe University, Ankara, 06100, Turkey

**Keywords:** bioadhesive film, cationic nanoparticle, core–shell nanoparticle, docetaxel, glioma

## Abstract

**Background:** Brain tumors are the most common tumors among adolescents. Although some chemotherapeutics are known to be effective against brain tumors based on cell culture studies, the same effect is not observed in clinical trials. For this reason, the development of drug delivery systems is important to treat brain tumors and prevent tumor recurrence. The aim of this study was to develop core–shell polymeric nanoparticles with positive charge by employing a chitosan coating. Additionally, an implantable formulation for the chemotherapeutic nanoparticles was developed as a bioadhesive film to be applied at the tumor site following surgical operation for brain glioma treatment. To obtain positively charged, implantable nanoparticles, the effects of preparation technique, chitosan coating concentration and presence of surfactants were evaluated to obtain optimal nanoparticles with a diameter of less than 100 nm and a net positive surface charge to facilitate cellular internalization of drug-loaded nanoparticles. Hydroxypropyl cellulose films were prepared to incorporate these nanoparticle dispersions to complete the implantable drug delivery system.

**Results:** The diameter of core–shell nanoparticles were in the range of 70–270 nm, depending on the preparation technique, polymer type and coating. Moreover, the chitosan coating significantly altered the surface charge of the nanoparticles to net positive values of +30 to +50 mV. The model drug docetaxel was successfully loaded into all particles, and the drug release rate from the nanoparticles was slowed down to 48 h by dispersing the nanoparticles in a hydroxypropyl cellulose film. Cell culture studies revealed that docetaxel-loaded nanoparticles cause higher cytotoxicity compared to the free docetaxel solution in DMSO.

**Conclusion:** Docetaxel-loaded nanoparticles dispersed in a bioadhesive film were shown to be suitable for application of chemotherapeutics directly to the action site during surgical operation. The system was found to release chemotherapeutics for several days at the tumor site and neighboring tissue. This can be suggested to result in a more effective brain tumor treatment when compared to chemotherapeutics administered as an intravenous bolus infusion.

## Introduction

A brain tumor is known as an abnormal growth of neoplastic cells within the brain or the central spinal canal. In the United States, it is estimated that 23,800 new cases and 16,700 deaths will occur in 2017 due to brain and other nervous system cancers. Brain and other nervous system cancers are the second most common tumor type from birth to the age of 19, thus having a high impact on public health and quality of life [1].

Surgical operation is the main treatment option for brain tumors; chemotherapy or radiotherapy are generally applied after surgery to remove remaining tumor cells and avoid the recurrence of the tumor [2–3]. At this stage, intravenous or orally administered chemotherapy drugs have very low efficacy due to challenges in reaching the brain and tumor area. The blood brain barrier (BBB) is the essential protection of the brain and only 1% of chemotherapeutic agents can pass this barrier without losing their pharmacological activity [4–9]. It is possible to bypass the BBB and reach the tumor site directly with implantable drug delivery systems such as Gliadel^®^, which is the chemotherapeutic drug carmustine-loaded wafer implant. These drug delivery systems can be implanted after surgical removal of the tumor, facilitating chemotherapy administration to prevent recurrence of the tumor at the time of tumor tissue removal by surgical operation.

Among the anticancer drugs that are used in clinics, the taxane family of drugs such as paclitaxel and docetaxel are known to be highly effective against a variety of cancer cells in vitro due to disruption of microtubule function. However, they are known to have severe solubility problems in aqueous media, therefore co-solvents or excipients are used to improve their solubility to facilitate injectable formulation development. Unfortunately, these solubilizing agents may often cause serious side effects. Thus, the necessity of a safe and effective formulation and drug delivery approach emerges for these potent anticancer drugs from the taxane family [10–13].

Successful treatment of brain cancer is dependent on the efficient and safe delivery of chemotherapeutic agents to the tumor site, while avoiding possible side effects. The development of novel drug delivery systems with reduced side effects is an important breakthrough and nanoparticles are promising in this field as they enable localized drug delivery to target sites and enhanced cellular uptake. Nanoparticle-based drug delivery systems can be prepared with synthetic and natural polymers. As an advantage, their surface properties can be modified to increase cellular penetration and prolonged drug release. Additionally, suitable nanoparticle-based drug delivery systems can bypass biological barriers or benefit from enhanced permeability and retention (EPR) effect thanks to their smaller size. They can also encapsulate hydrophobic drugs as their cargo to improve solubility at the target site. Consequently, nanoparticle-based drug delivery systems can protect drug activities in biological systems and allow targeted drug delivery [14–17].

Polycaprolactone (PCL) is a synthetic hydrophobic polymer, which is prepared by ring opening polymerization of the monomer ε-caprolactone. It is used as a polymer in preparation of nanoparticles and other drug depot and delivery systems. Moreover, PCL is reported to be nontoxic, biocompatible and biodegradable and is approved for therapeutic use in humans by the FDA. PCL can be copolymerized with other synthetic polymers such as polyethylene glycol (PEG) and polyethylene oxide (PEO) to obtain new polycaprolactone derivatives with various novel properties [18–19]. There are several studies reported on PCL as a functional excipients for the preparation of nanoparticulate drug delivery systems with favorable drug loading and release characteristics for hydrophobic anticancer molecules in particular [18–21]. However, the application of core–shell PCL nanoparticles to tumor targeting with docetaxel on a glioma model is very rare. Recently, active-targeted docetaxel-loaded PEG/PCL nanoparticles were prepared successfully for glioblastoma therapy by Gao et al. Cellular uptake and tumor spheroid uptake studies on U87 human glioma cells show that active targeted PEG/PCL nanoparticles enhanced tumor penetration [22]. Besides that, Ungaro et al. obtained docetaxel-loaded core–shell PEO/PCL nanoassemblies for passive targeting of the anticancer drug to cancer cells. Their results showed that docetaxel-loaded PEO/PCL nanoparticles were more effective on growth inhibition of breast and prostate cancer cells when compared to free docetaxel [23]. Core–shell nanoparticles are also used as non-viral vectors for the treatment of glioma. Zamora et al. prepared photochemical internalization mediated polyamine core–shell nanoparticles for tumor suppressor gene delivery. Their results showed that the prepared nanoparticles enhanced the delivery of tumor suppressor genes on U87 and U251 glioma cells [24]. Wang et al. used core–shell nanoparticles for drug and gene co-delivery. They prepared magnetic PLGA/polymeric liposome carriers to achieve sustained release of the model drug epidoxorubicin as carriers of pEGFP DNA complexes. The results demonstrated that co-delivery of drug and gene could be performed and strong inhibition effects on glioblastoma can be achieved with their system [25]. Additionally, magnetic core–shell nanoparticles have been studied for targeting and delivery of chemotherapeutic drugs for glioma treatment. Fang et al. prepared core–shell nanocapsules for co-delivery of the hydrophilic drug doxorubicin, and the hydrophobic drug curcumin. Their results showed that the synergistic cytotoxic effect on RG2 glioma cells was obtained by dual drug targeting. Besides that, the magnetic and ligand targeting resulted in elevated cellular uptake of nanocapsules in glioma treatment [26]. Yang et al. successfully obtained targeted and traceable core–shell nanoparticles for carmustine (BCNU) delivery. These systems prolonged the half-time and also enhanced the concentration of BCNU in the brain tumor area [27]. In addition to drug delivery, core–shell nanoparticles such as magnetic nanoparticles [28], quantum dots [29], nanodiamonds [30], nanocrystals [31] and iron oxide nanoparticles [32] are studied as imaging and detection agents of glioma.

An interesting, biocompatible and simple approach is to coat the nanoparticles with cationic polymers to enhance cellular penetration and prolong retention at biological membranes. Cationic nanoparticles are able to pass through biological membranes with facilitated uptake by cells, due to their strong cellular interaction with negatively charged biological membranes. Another important advantage is that they can mask the negative charge of anionic drugs to escape the mononuclear phagocytic system (MPS). Ionic particles can be easily determined by the MPS, therefore drug-loaded particles (which have neutral or near-neutral surface charge) are more prone to escape from the MPS. Cationic nanoparticles can also condense nucleic acid (DNA, RNA) or proteins to form polyplexes for intracellular gene/drug delivery. In this context, chitosan (CS) is used as a positively charged coating polymer with optimal results. CS, which is produced commercially by deacetylation of chitin, is a linear polysaccharide. It is a biocompatible and nontoxic natural polymer [33–39] which is known to act as a penetration enhancer, mucoadhesive [40], antitumor [41] and immune-adjuvant [42], which contribute to the potential of this biopolymer for drug delivery and formulation.

Although systemic application is frequently preferred for nanomedicines, local administration is a major opportunity when on-site therapy is possible and intended for. In fact, local or implantable administration for therapeutic nanoparticles help reduce systemic side effects, bypass BBB and improve efficacy of the drug by forming a constant drug reservoir directly at target site [43–49].

The goal of this study was to evaluate and characterize implantable cationic nanoparticle-loaded film formulations as post-surgical local delivery systems for docetaxel (DOC). PCL and its derivative poly(ethylene glycol)-block-poly(ε−caprolactone) methyl ether (mePEG-PCL) were used to prepare these nanoparticles by the nanoprecipitation technique with surface modification by coating with CS. The nanoparticles were administered as a dispersion in the hydroxypropylcellulose (HpC) Klucel™ bioadhesive film. The aim was to develop an implantable, local nanomedicine capable of prolonged release at the tumor site to create a drug reservoir after surgical removal of glioma, avoiding progression and recurrence of the tumor by killing cancer cells in surrounding tissues.

## Results and Discussion

### Pre-formulation studies

Pre-formulation studies were evaluated to select optimal nanoparticle formulations. Particle size, polydispersity index and surface charge are known to be critical parameters that significantly affect cellular uptake, interaction with biological membranes, absorption rate, biodistribution in the body, as well as the physical stability of the nanoparticles [50]. It is known that nanoparticles can escape from systemic circulation via fenestrations, which are small openings through the endothelial barrier. The size of these fenestrations depends on the type of organ and tumor [51]. For this reason, the nanoparticle particle size is crucial for a targeted organ/tumor.

As core–shell polymeric nanoparticles can be prepared using different techniques, the optimal preparation technique was determined to obtain smaller, monodisperse nanoparticles with favorable stability. Three different preparation techniques, emulsion/solvent evaporation, double emulsion and nanoprecipitation, were used to prepare PCL or mePEG-PCL nanoparticles.

As seen in Table 1, the mean diameter of PCL nanoparticles was found to be 160–350 nm. It was clearly shown that the preparation technique significantly affects the particle size (*p* < 0.05). In addition, the polydispersity index of the PCL nanoparticles also depends on the preparation technique, directly. Studies showed that PCL nanoparticles which were prepared by emulsion-based techniques have larger diameters, especially in the case of the double emulsification technique when compared to nanoprecipitation. These results shows compare well with the literature [52–55]. According to the data in Table 1, significantly smaller nanoparticles were obtained with mePEG-PCL (*p* < 0.05). The preparation method had a similar effect on mePEG-PCL nanoparticles as well.

**Table 1:** The effect of different preparation methods on physicochemical properties of blank PCL and mePEG-PCL nanoparticles (*n* = 3 ± SD).

		Mean diameter ± SD (nm)	PDI ± SD	Zeta potential ± SD (mV)

PCL nanoparticles	nanoprecipitation	168 ± 3	0.10 ± 0.02	−17 ± 0.4
	emulsification/solvent evaporation	184 ± 3	0.29 ± 0.4	−18 ± 0.8
	double emulsion	352 ± 2	0.39 ± 0.02	−8 ± 0.1
mePEG-PCL nanoparticles	nanoprecipitation	77 ± 3	0.17 ± 0.04	−13 ± 3.2
	emulsification/solvent evaporation	146 ± 3	0.27 ± 0.004	−19 ± 1.3
	double emulsion	170 ± 2	0.19 ± 0.01	−5 ± 0.24

The double emulsion method yielded the largest particle size and polydispersity index for blank PCL and mePEG-PCL nanoparticles. The double emulsion method involves two emulsification steps. For this reason, the particle size increases in each emulsification step. In addition, double emulsion resulted in a significant difference in the zeta potential of nanoparticles (*p* < 0.05). The surface charge of blank nanoparticles prepared by double emulsification was closer to neutral charge as compared to those prepared by the nanoprecipitation or emulsification/solvent evaporation methods.

Our results clearly show that mePEG-PCL nanoparticles have significantly smaller particle size than PCL nanoparticles for all preparation techniques (*p* < 0.05). In the literature, mePEG-PCL nanoparticles prepared by nanoprecipitation have been found to be generally smaller than 120 nm [53,56–58]; however, PCL nanoparticles prepared by the same technique are between 200–300 nm [55,59]. mePEG-PCL can be solubilized in organic solvents more easily, thanks to the hydrophilic PEG chains as compared to PCL. This difference may be effective for the spontaneous formation of nanoparticles at the interface and at obtaining a smaller particle size.

Another important parameter affecting the final nanoparticle properties is reported to be the presence and concentration of the surfactant, which can influence particle size distribution and surface properties. According to the results in Table 2, the addition of surfactant did not reduce the particle size; on the contrary, the mean particle size significantly increased proportional to the concentration of PF68 for both polymer PCL and mePEG-PCL (*p* < 0.05). Although it has been shown in literature that addition of surfactant causes increased solubility of polymer in aqueous media and decreases the particle size [60], the exact opposite of this situation has been found, too [61]. In our studies, the addition of surfactant for both nanoparticle formulations may have led to the formation of an extra surfactant layer and this layer increases the particle size. Besides that, this surfactant layer probably covered the polymer surface and thus the zeta potential of the nanoparticles approached a more neutral value.

**Table 2:** The effect of different preparation methods on the physicochemical properties of blank PCL and mePEG-PCL nanoparticles (*n* = 3 ± SD).

	PF68 concentration (v/v, %)	Mean diameter ± SD (nm)	PDI ± SD	Zeta potential ± SD (mV)

PCL nanoparticles	0	150 ± 0.5	0.08 ± 1.9	−22 ± 0.009
	0.5	163 ± 0.5	0.10 ± 0.5	−20 ± 0.02
	2	194 ± 0.8	0.09 ± 0.4	−15 ± 0.006
mePEG-PCL nanoparticles	0	71 ± 0.8	0.22 ± 0.004	−22 ± 1.9
	0.5	95 ± 3.9	0.50 ± 0.03	−27 ± 2.1
	2	92 ± 1.4	0.31 ± 0.04	−20 ± 3.8

To render a positive surface charge to blank PCL or mePEG-PCL nanoparticles, chitosan was incorporated as a cationic coating polymer. The mean particle size increased with increasing CS concentration, as can be expected due to the thicker coating layer (Table 3), as has been similarly demonstrated in the literature [62–65]. CS changed the surface charge from −19 to +39 mV and further to +53 mV by increasing the concentration of CS in the PCL nanoparticle formulations. In addition, the surface charge of mePEG-PCL nanoparticles significantly increased up to 31 mV, depending on the CS concentration (*p* < 0.05), directly. Chitosan-modified core–shell nanoparticles were studied for glioma therapy by Qian et al*.* where a PLGA nanoparticle surface was modified with CS and cellular uptake of nanoparticles was determined. They showed that cellular uptake is related to chitosan concentration and particle size. According to their results, chitosan modification increased the particle size and decreased the cellular uptake of nanoparticles [66]. Cationic core–shell nanoparticles are also quite suitable for the delivery of negatively charged gene and drug to tumor tissue. Wei et al. used cationic core–shell nanoparticles for the active targeted delivery of siRNA on an intracranial U87 glioma model. They demonstrated that active targeted and cationic core–shell nanoparticles could be effective in inhibition of tumor proliferation with higher accumulation in tumor area when they are administered intravenously [67]. Different studies also showed that nanoparticles that have a zeta potential value smaller than 30 are more stable and show reduced aggregation [68–69].

**Table 3:** The effect of chitosan concentration on the physicochemical properties of blank PCL and mePEG-PCL nanoparticles (*n* = 3 ± SD).

	Chitosan concentration (wt/v, %)	Particle size ± SD (nm)	PDI ± SD	Zeta potential ± SD (mV)

PCL nanoparticles	0	170 ± 0.1	0.07 ± 0.02	−20 ± 0.6
	0.01	196 ± 14	0.25 ± 0.03	39 ± 0.9
	0.025	218 ± 9	0.20 ± 0.02	54 ± 1.9
mePEG-PCL nanoparticles	0	71 ± 0.8	0.22 ± 0.004	−22 ± 1.9
	0.01	120 ± 2	0.39 ± 0.006	31 ± 1.8
	0.025	155 ± 1.6	0.42 ± 0.02	31 ± 1.3

### In vitro characterization of docetaxel-loaded nanoparticles

**Figure 1:**
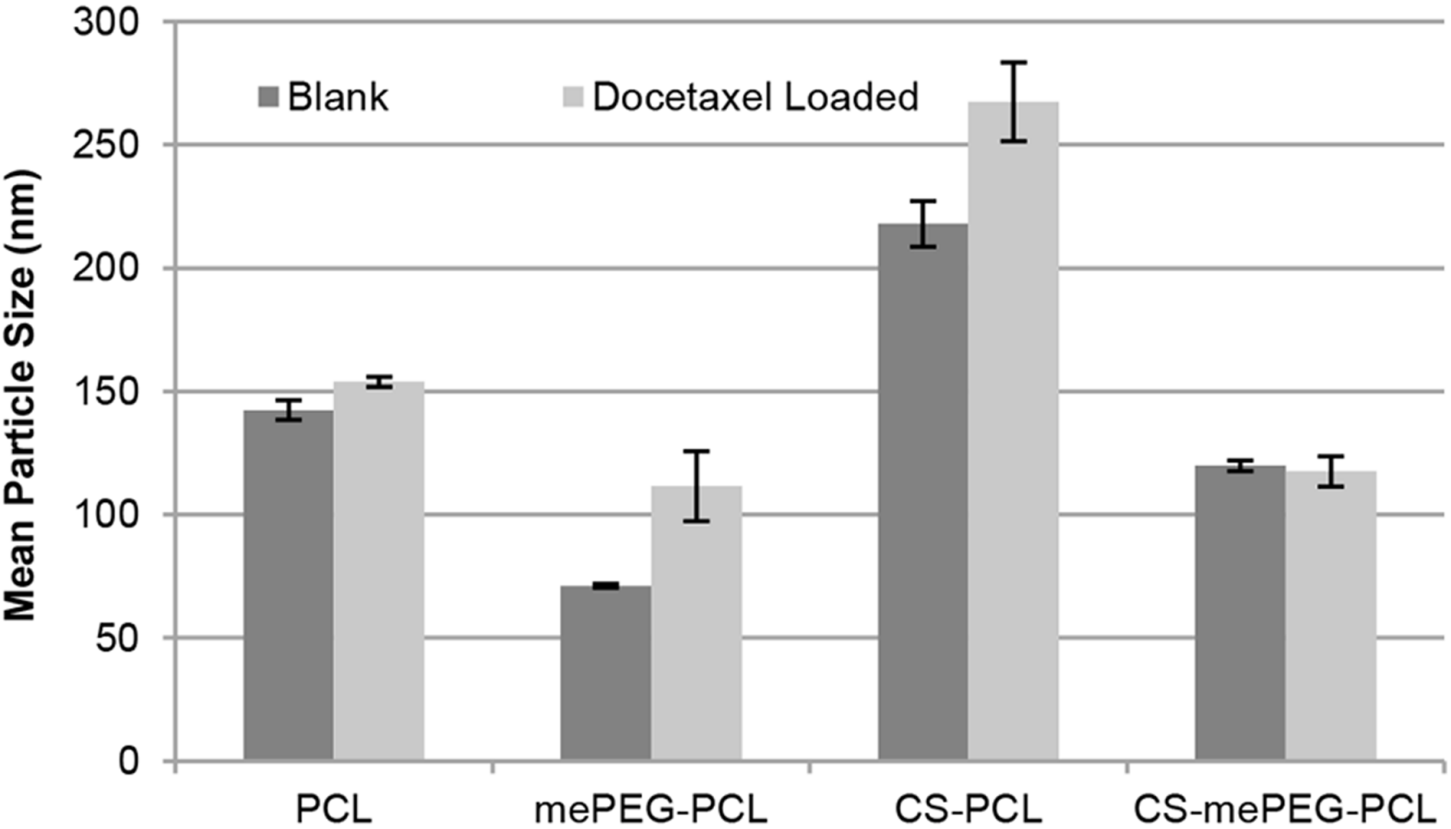
Particle size of blank and drug-loaded nanoparticles (*n* = 3, ± SD).

**Figure 2:**
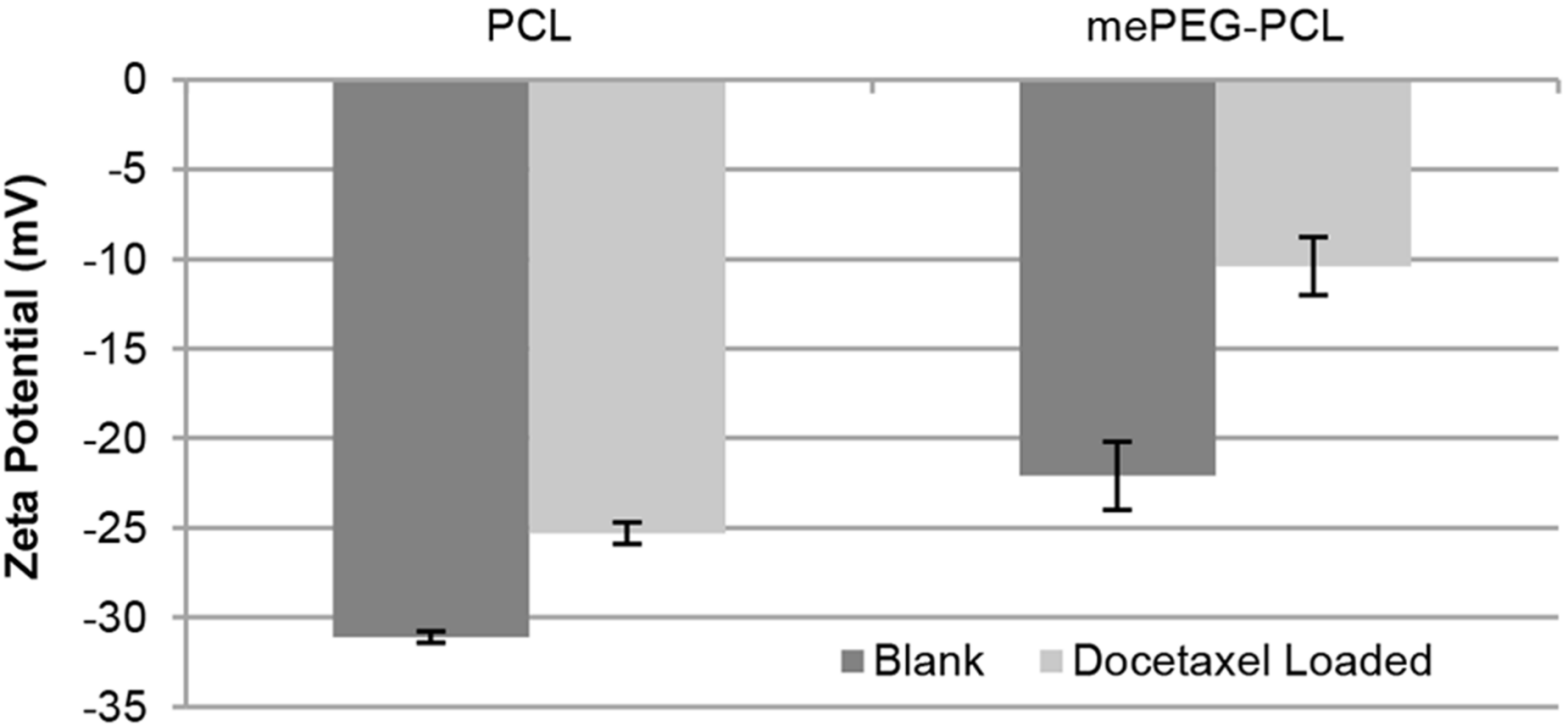
Zeta potential of blank and drug-loaded nanoparticles (*n* = 3, ± SD).

According to the results of the pre-formulation studies, the final formulation parameters were determined and nanoparticles were prepared by the nanoprecipitation technique without surfactant due to their smaller particle size and polydispersity index. In order to render the surface charge positive, 0.01% wt/v chitosan was added to the aqueous phase. This concentration was selected since nanoparticles that have zeta potential outside the range of ±30 mV are known to be prone to aggregation [54–55]. For PCL nanoparticles, the drug amount was set at 10% of the PCL weight [60,63,70]. Therefore, DOC (0.01% w/v) was added in the organic phase with the polymer in the nanoprecipitation technique for both polymers. The particle size and zeta potential of nanoparticles for PCL or mePEG-PCL nanoparticles are shown in Figure 1 and Figure 2. CS coating and drug loading causes an increase in particle size, as expected. According to our results, the drug-loaded nanoparticle diameter is generally 10 to 50 nm higher than the unloaded nanoparticles.

### Physical stability of nanoparticles

**Figure 3:**
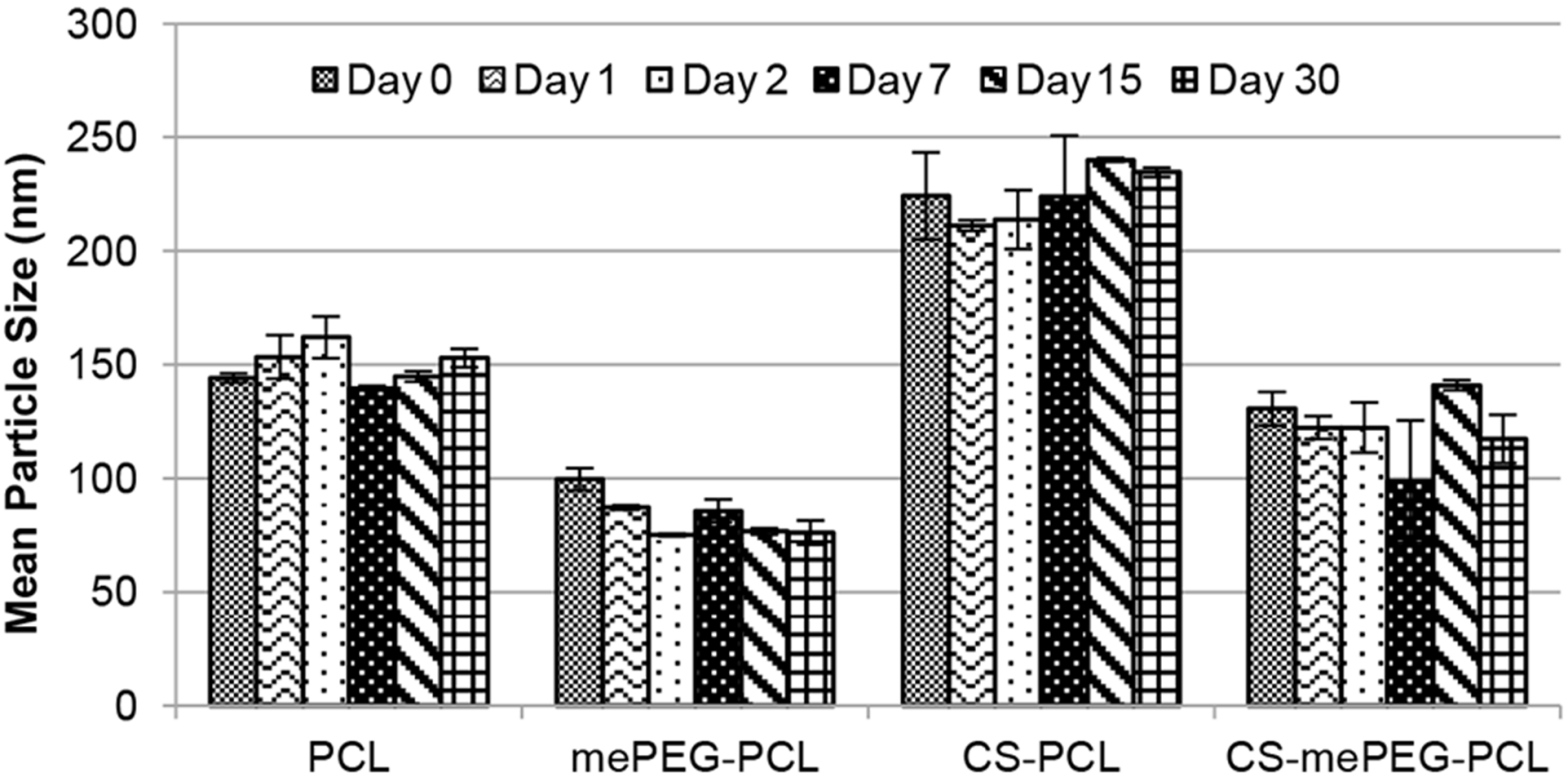
Mean particle diameter of nanoparticle formulations over the course of 30 days (*n* = 3, ± SD).

The physicochemical properties of all formulations have been monitored to investigate their physical stability in aqueous medium for 30 days; the results are shown in Figure 3. The diameter of anionic and cationic PCL nanoparticles increased by 8–10 nm and mePEG-PCL nanoparticles increased by 13–23 nm during this period. However, this increase is not statistically significant. Consequently, it can be said that aqueous dispersions of drug-loaded nanoparticles are physically stable for a period of 1 month.

### Encapsulation efficacy of drug-loaded nanoparticles

The docetaxel concentration in nanoparticle formulations was directly quantified with a validated HPLC method and expressed in terms of associated drug (%). In the literature, the encapsulation efficacy for PCL nanoparticles was found to be between 65−71% [60,70] and for mePEG-PCL nanoparticles to be 80−90% [56,58]. According to our results, the encapsulation efficacy of mePEG-PCL nanoparticles was not found to be as high as reported in the literature. This may be caused by the differences in the molecular weight of the PCL used in mePEG-PCL.

**Figure 4:**
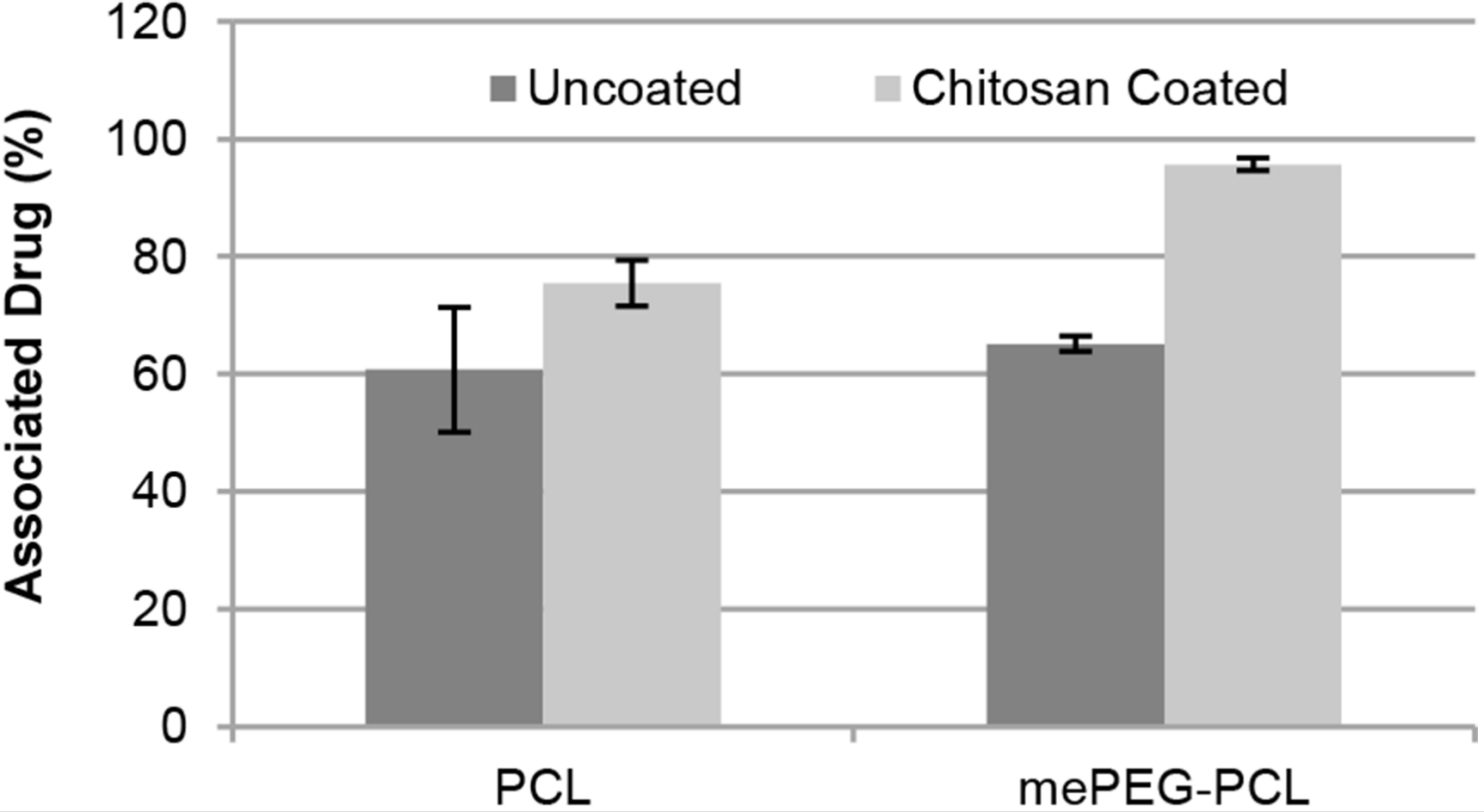
Docetaxel encapsulation efficiency of nanoparticle formulations (*n* = 3, ± SD).

The zeta potential of DOC solutions was measured as −14 mV and the encapsulation efficacy was significantly improved for both PCL and mePEG-PCL nanoparticles by coating with the cationic polymer CS, as shown in Figure 4. This is attributed to the strong electrostatic interaction between the anionic drug docetaxel with the cationic coating. The encapsulation efficacy of PCL and mePEG-PCL nanoparticles is not significantly different from one other (*p* > 0.05) but CS-coated mePEG-PCL nanoparticles have the largest encapsulation efficacy (*p* < 0.05). The mePEG-PCL polymer is more hydrophilic than PCL, as previously mentioned, and this property may be effective for the high encapsulation efficacy as well as smaller particle size as shown in the literature [56,58].

### In vitro release studies

**Figure 5:**
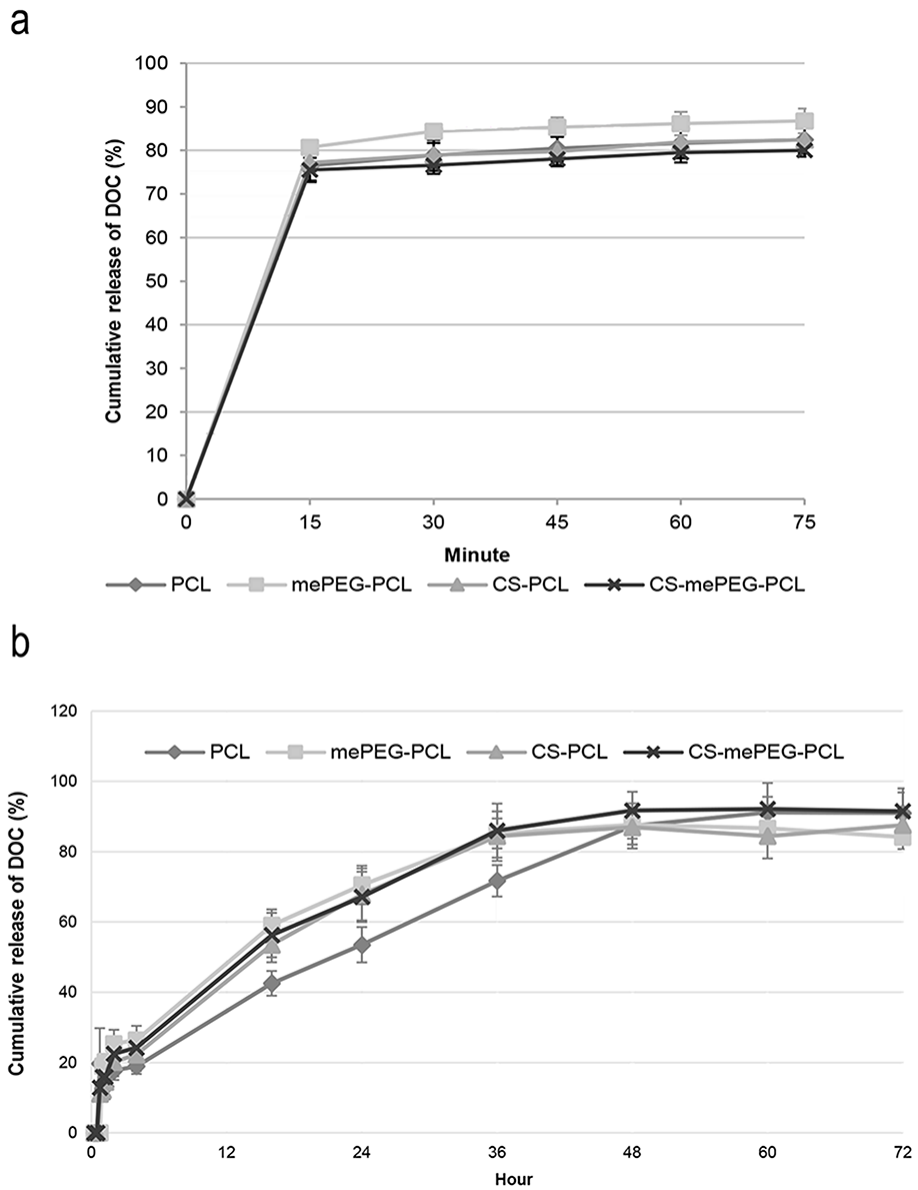
Cumulative release profile of DOC from nanoparticles (a) and nanoparticulate DOC from HpC films (b) (*n* = 3, ± SD).

The in vitro release profile of docetaxel from nanoparticle dispersion and nanoparticle-loaded HpC films was determined using the dialysis bag method in PBS pH 7.4 with HPLC. As seen in Figure 5, DOC was completely released from all nanoparticles within 1 h. The PCL nanoparticles are generally expected to give a longer release time due to slower degradation time of PCL [56,58,60] if the drug is entrapped in a nanoparticle matrix. In our study, it is suggested by the encapsulation data shown in Figure 4 that DOC is largely adsorbed onto the coating layer and therefore released rapidly by desorption of the drug from the nanoparticle surface. The slower release was achieved by loading the DOC nanoparticles into a HpC film. By examining the release profiles of DOC from nanoparticle-loaded HpC film, it can be seen that 50% DOC was released in the first 16 h and complete release of the encapsulated drug was found to occur after 48 h with a slower rate (Figure 5). The structure of the HpC film may be effective in slowing the release. The release of water-insoluble drugs from HpC films was examined by different study groups and the release profile was shown to be completed within approximately 10 h [71–73]. In another study regarding the release of paclitaxel (which is another member of taxane class, such as docetaxel, released from nanocomposite film) the initial release was observed within 7 h due to the rapid release of drugs from surface of the film [74]. Our studies proved that the DOC-encapsulated PCL-nanoparticle-loaded film formulation is quite suitable to provide a drug reservoir after surgical removal of glioma to avoid progression recurrence during the first 2 days.

### Cell culture studies

#### Cytotoxicity assay for blank nanoparticles

**Figure 6:**
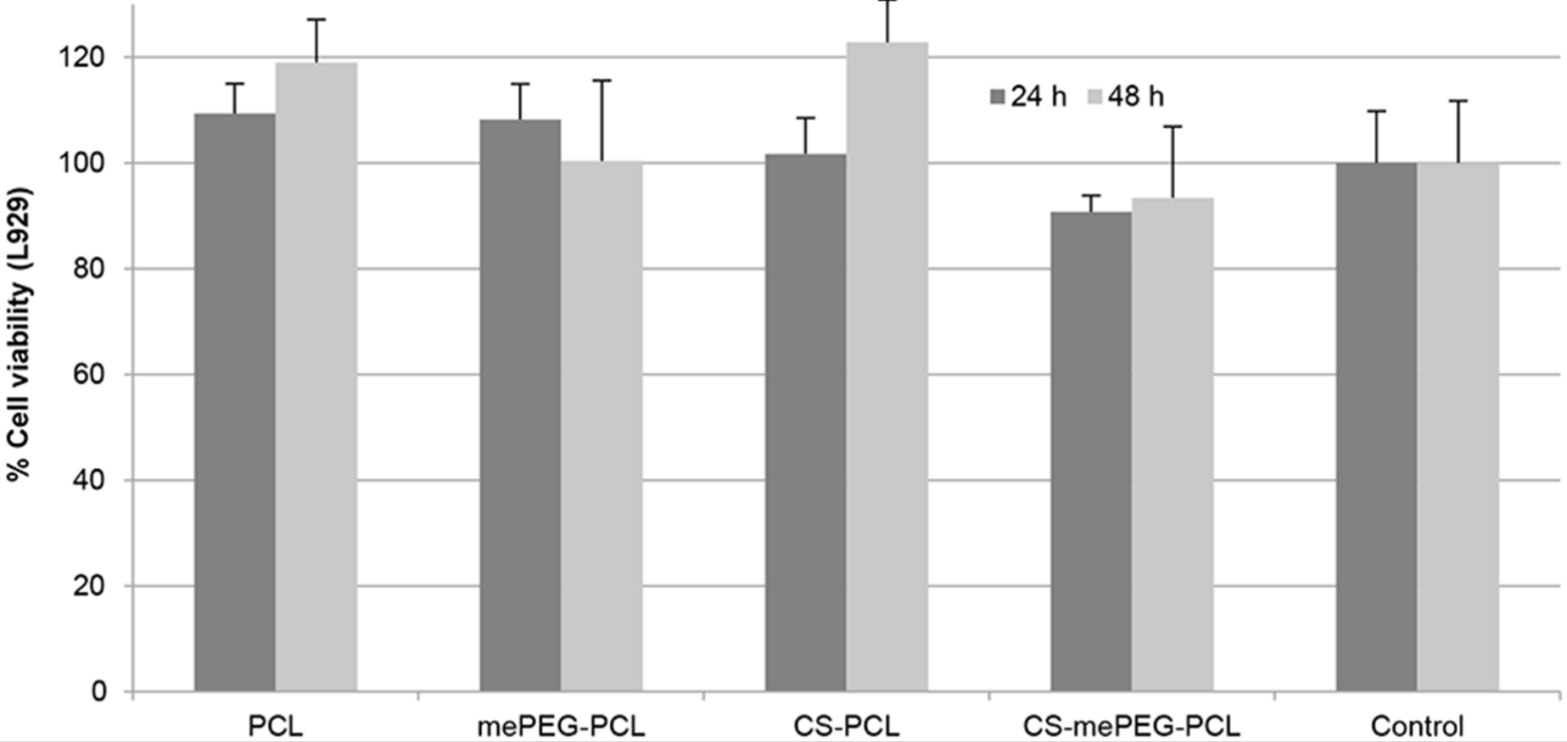
Cell viability of blank nanoparticles for 24 and 48 h (*n* = 3, ± SD).

Mouse fibroblast cell lines L929 (recommended by the USP for the cytotoxicity evaluation of polymeric systems) were used to determine the cytotoxicity of blank nanoparticles with MTT assay. According to MTT assay, cell viability for L929 cells is given in Figure 6 for 24 h and 48 h. When compared with the control group, the blank formulations were found to have no cytotoxic effect on L929 fibroblast cells (the differences between groups were statistically insignificant, *p* > 0.05), and it can be suggested that all formulations are safe for in vivo application, regardless of dose or time.

#### Anticancer efficacy of docetaxel-loaded nanoparticles

**Figure 7:**
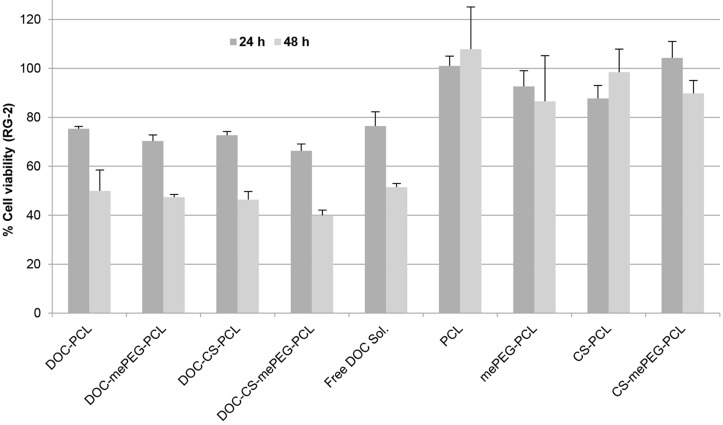
RG2 cell viability with blank and DOC-loaded nanoparticles for 24 and 48 h (*n* = 3, ± SD).

The anticancer efficacy of drug-loaded nanoparticle dispersions were determined on rat glioma cell line RG2. As seen in Figure 7, anticancer efficiency is enhanced for DOC both with time-dependent and formulation-dependent mechanisms. The cell culture data showed that DOC-loaded CS-mePEG-PCL nanoparticle dispersions have a significantly higher cytotoxic effect than DOC solutions in DMSO (*p* < 0.05). Besides, blank nanoparticle formulations did not exert any toxic effect on RG2 cells. As a result, CS-coated nanoparticle formulations were found to be significantly more effective against glioma cells than nanoparticles that have negative surface charge (*p* < 0.05). Cationic nanoparticles may interact and pass the cell membrane more easily due to their opposite electrical charge with respect to the cell surface. On the other hand, it is known that chitosan also possesses intrinsic antitumor activity due to activation of the caspase-3 mechanism [41]. This may explain the synergistic mechanism of why chitosan-coated nanoparticles are more effective on cancer cells when compared with non-coated nanoparticles.

## Conclusion

In this study, the anticancer drug DOC, encapsulated in anionic and cationic polymeric nanoparticles and administered in a bioadhesive film formulation, was successfully developed to apply the chemotherapeutic drug directly to the action site after surgical operation of glioma treatment. All formulations were characterized in terms of mean particle size, polydispersity index, zeta potential, drug loading capacity, drug release profile and cytotoxicity. When nanoparticle formulations are compared with each other, mePEG-PCL nanoparticles have a significantly smaller particle size. Furthermore, drug loading and anticancer efficacy in rat glioma cells were drastically increased by cationic coated with CS. Thus, mePEG-PCL and CS-coated mePEG-PCL nanoparticle formulations can be used for further studies. Moreover, the release profile was prolonged by up to 2 days due to the implantable film formulation. This result could be a solution to the premature drug release and dose dumping known to occur with the use of nanoparticles. In the light of the cell culture data, all nanoparticle formulations increased the anticancer effects of DOC in free form, while blank nanoparticles were found to be nontoxic on L929 and RG-2 cell lines. It can be said that all drug-loaded nanoparticles are biocompatible, safe and effective against glioma.

Our study emphasizes that polycaprolactone and PEGylated derivatives are suitable for the development of nanoparticles and their zeta potential can be varied with chitosan coating. When further loaded into films, these nanoparticles seem to be a potential drug delivery system for docetaxel for glioma treatment and a good candidate for further evaluation in animal studies. This film formulation can be implanted after surgical removal of a tumor and provide a drug reservoir after surgical removal of glioma during the initial days to avoid progression and recurrence by killing cancer cells in neighboring tissue.

## Experimental

### Materials

PCL (*M*_W_: 80,000 Da) and mePEG-PCL (PEG:PCL *M*_W_: 5,000:5,000 Da) were purchased from Sigma-Aldrich, USA. Chitosan (Protasan^®^ G 113, *M*_W_ < 200 kDa, deacetylation degree 75–90%) was purchased from FMC Biopolymers, Norway. HpC (Klucel™ hydroxypropylcellulose) was purchased from Ashland, USA. The model anticancer drug, docetaxel (purity 97%), was purchased from Fluka, Switzerland. Dialysis tubing cellulose membrane (average flat width 25 mm, MWCO: 14,000 Da) and all organic solvents and chemicals were purchased from Sigma-Aldrich, Germany. Ultrapure water was obtained from a Millipore Simplicity 185 ultrapure water system, France and used without further purification.

### Methods

#### Pre-formulation studies

Pre-formulation studies were carried out to optimize the final nanoparticle physical properties. The formulation and technological variables that are known to influence the nanoparticle properties were evaluated. Primarily, different nanoparticle preparation techniques were used to prepare the nanoparticles, which was then followed by varying formulation parameters such as surfactant concentration and coating polymer concentration, as summarized in Table 4.

**Table 4:** Pre-formulation parameters for nanoparticle preparation.

preparation technique	nanoprecipitation
	emulsion/solvent evaporation
	double emulsion
polymer molecular weight (Da)	mePEG-PCL (*M*_W_: 5000:5000)
	PCL (*M*_W_: 80,000)
surfactant (PF68) concentration (% v/v)	0
	0.5
	2
coating polymer chitosan amount (% w/v)	0
	0.01
	0.025

As different preparation techniques drastically affect the nanoparticle size and degree of drug interaction, several well-established nanoparticle preparation methods were evaluated for PCL and mePEG-PCL nanoparticles. These preparation methods can briefly be summarized as follows.

Nanoprecipitation: The polymer (PCL or MePEG-PCL) was dissolved in acetone (0.1% v/w) under moderate heating. This organic solution was added to ultrapure water (1:2 v/v) dropwise under magnetic stirring at room temperature. As a result, nanoparticles were spontaneously obtained. The organic solvent was then evaporated under vacuum at 40 °C. The formulations were filtered through a 0.45 μm pore membrane filter to eliminate polymer aggregates. Cationic-coated nanoparticles were obtained with the same technique with the minor difference that CS was dissolved in ultrapure water to form the aqueous phase during preparation.

Emulsion solvent/evaporation: The polymer (PCL or MePEG-PCL) was dissolved in dichloromethane (0.1% v/w) under magnetic stirring. This organic phase (5 mL) was added to ultrapure water (20 mL) containing PF68 (1% v/w) and polyvinyl alcohol (0.1% v/w) and emulsified by ultraturrax at 13,000 rpm. The organic solvent was evaporated under vacuum at 40 °C. The formulations were filtered through a 0.45 μm pore filter to eliminate polymer aggregates.

Double emulsion: PF68 (1% w/v) was dissolved in ultrapure water (1 mL) and the polymer (PCL or MePEG-PCL) (0.5% w/v) was dissolved in dichloromethane (5 mL) under magnetic stirring. Ultrapure water containing PF68 (1% w/v) was added to the organic solution containing polymer. This mix was emulsified by ultraturrax at 13,000 rpm. This primary emulsion was added to 20 mL ultrapure water containing PF68 (1% w/v) and polyvinyl alcohol (0.1% w/v) and emulsified by ultraturrax at 13,000 rpm. The organic solvent was evaporated under vacuum at 40 °C. The formulations were filtered through a 0.45 μm pore sized filter to eliminate polymer aggregates.

#### Preparation of nanoparticle-loaded film formulations

Following the selection of optimal nanoparticle formulations, docetaxel-loaded nanoparticles were loaded into film formulations to prolong the activity of the nanoparticles at the administration site. Briefly, HpC (Klucel™) was dissolved in ultrapure water (5% w/v). The lyophilized nanoparticle powder was added to this mix and stirred. This solution was slowly poured on a water-impermeable polyethylene terephthalate (PET) film and dried at room temperature for 48 h. Finally, the HpC film was removed from the surface of the PET film to obtain the final product.

#### Nanoparticle characterization

Particle size distribution and surface charge analysis: the mean particle diameter and polydispersity index of the nanoparticles were determined by dynamic light scattering (DLS) technique using a Malvern NanoZS (Malvern Instruments, UK). All formulations were measured at a scattering angle of 173° at a temperature 25 °C (*n* = 3). The surface charge of the nanoparticles was determined by using a disposable capillary cell with the Malvern Zetasizer Nano ZS at room temperature (*n* = 3).

Physical stability upon storage: The physical stability of the nanoparticles was determined by repeated measurement and comparison of the particle size, polydispersity index and zeta potential data for 30 days at specific time intervals. During this time, the formulations were stored as aqueous dispersions in ultrapure water at +4 °C.

Encapsulation efficiency: DOC encapsulation of nanoparticle formulations were determined directly with validated HPLC method by using an HP Agilent 1100 instrument. The HPLC system consisted of a reverse phase Develosil ODS-UG-5 (4.6 mm/150 mm 5.6 μm) column and acetonitrile/water (50:50 v/v) as mobile phase delivered at a flow rate of 1.00 mL/min. A 50 µL injection volume was used for analysis. The DOC was quantified by a UV detector set at λ = 229.6 nm at 25 °C. Drug loading was expressed as associated drug percentage, quantifying the drug quantity bound to nanoparticles. The associated drug percentage (%) was calculated as follows:


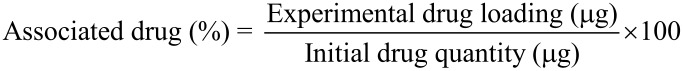


In vitro docetaxel release: The in vitro release profile of DOC from nanoparticles and film formulations was determined by using the dialysis membrane technique under sink conditions in a shaking water bath at 37 °C in phosphate buffer solution (PBS) pH 7.4. Briefly, the drug-loaded nanoparticle dispersions or 1 cm^2^ film were added in dialysis membrane (Cellulose Membrane MWCO: 14,000 Da, Sigma-Aldrich, Germany) and closed with stoppers. This bag was placed in PBS pH 7.4 containing 0.1% Tween 80 to provide sink conditions. Samples were taken from the PBS at specific time intervals and the released DOC amount was determined directly with validated HPLC method.

#### Cell culture studies

Cytotoxicity assay for blank nanoparticles and drug-loaded nanoparticles: Mouse fibroblast cells L929 were used to determine the cytotoxicity of blank nanoparticles as this is defined as a standard method for cytotoxicity determination by United States Pharmacopoeia. After the cytotoxicity testing of blank nanoparticles, rat glioma cells RG2 were used to determine the anticancer activity of docetaxel (500 nM) incorporated nanoparticles. The cell lines were cultured as a monolayer in Dulbecco’s modified Eagle’s medium (DMEM) supplemented with 10% fetal bovine serum (FBS), penicillin (100 units/mL) and streptomycin (100 µg/mL) and maintained at 37 °C in a humidified 5% CO_2_ incubator. The cells were seeded in 96-well tissue culture and incubated for 24 and 48 h. Then, DMEM was replaced with fresh medium containing blank nanoparticle formulations and incubated for 48 h. MTT assay was applied to determine cell viability. 20 µL of MTT solution in PBS (5 mg/mL) were added in each well and incubated for 4 h. 80 µL of MTT lysis solution containing SDS (23% w/v) and DMF (45% v/v) in ultrapure water were added in plates and incubated overnight. The optical density (OD) was determined by a microplate reader (Molecular Devices, USA) at 450 nm (*n* = 3). The results were expressed in terms of cell viability (%) according to the equation:


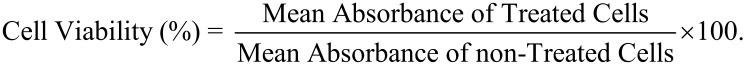


#### Statistical analysis

All statistical analyses were performed by Student’s *t*-test. *p* < 0.05 was considered to denote a statistically significant difference.
